# “Health Comes First”: Action Tendencies to Health-Related Stimuli in People with Health-Anxiety as Revealed by an Emotional Go/No-Go Task

**DOI:** 10.3390/ijerph18179104

**Published:** 2021-08-28

**Authors:** Laura Sagliano, Raffaele Nappo, Mario Liotti, Mariarosaria Fiorenza, Chiara Gargiulo, Luigi Trojano, Massimiliano Conson

**Affiliations:** 1 Department of Psychology, University of Campania-Luigi Vanvitelli, 81100 Caserta, Italy; laura.sagliano@unicampania.it (L.S.); fy1@hotmail.it (R.N.); mariaros.fiorenza@gmail.com (M.F.); chiara.gargiulo21@gmail.com (C.G.); luigi.trojano@unicampania.it (L.T.); 2 Neapolisanit Rehabilitation Center, Via Funari, 80044 Ottaviano, Naples, Italy; 3 Department of Developmental and Social Psychology and Padua Neuroscience Center, University of Padua, 35100 Padua, Italy; mario.liotti@gmail.com

**Keywords:** health anxiety, attentional bias, anxiety sensitivity, health-related concerns, go/no-go task, motor inhibition

## Abstract

The processing of health-related stimuli can be biased by health anxiety and anxiety sensitivity but, at the moment, it is far from clear whether health-related stimuli can affect motor readiness or the ability to inhibit action. In this preliminary study, we assessed whether different levels of health anxiety and anxiety sensitivity affect disposition to action in response to positive and negative health-related stimuli in non-clinical individuals. An emotional go/no-go task was devised to test action disposition in response to positive (wellness-related), and negative (disease-related) stimuli in non-clinical participants who also underwent well-validated self-report measures of health anxiety and anxiety sensitivity. The main results showed that both health anxiety and anxiety sensitivity biased participants’ responses. Importantly, safety-seeking and avoidance behaviors differently affected action disposition in response to positive and negative stimuli. These preliminary results support the idea that health anxiety and anxiety sensitivity could determine a hypervigilance for health-related information with a different perturbation of response control depending on the valence of the stimuli. Health anxiety and health anxiety disorder do form a continuum; thus, capturing different action tendencies to health-related stimuli could represent a valuable complementary tool to detect processing biases in persons who might develop a clinical condition.

## 1. Introduction

Health anxiety is a form of anxiety characterized by an excessive worry about one’s own health and by a catastrophic interpretation of body sensations. It refers to a dimensional construct [1,2], ranging from mild expressions of fear to clinically significant concerns, as in the case of Illness Anxiety Disorder [3], formerly defined as hypochondriasis [4]. 

Previous studies demonstrated that health anxiety modulates the processing of health-related stimuli. In detail, individuals with elevated health anxiety show increased attentional interference by task-irrelevant health-threat information [5,6]. This attentional interference is highly specific for self-relevant threatening stimuli [5,7]. For instance, Gropalis et al. [7] investigated attentional and memory biases for health-related words (illnesses, bodily complaints, and panic-related words) in three health-related disorders: illness anxiety disorder, other somatoform disorders, and panic disorder. Results showed significant attentional biases toward all health-related word categories in patients with illness anxiety disorder, whereas a bias for panic-related words was found in the panic disorder group only. A few studies also investigated attentional biases for health-related stimuli in healthy individuals with high levels of anxiety sensitivity, i.e., the fear of anxiety-related sensations [8]. Employing a visual probe task, Lees, Mogg and Bradley [9] reported greater initial attentional bias to health-related pictures, but not to words, in people with high levels of anxiety sensitivity, compared to those with low levels of anxiety sensitivity. Taake, Jasper-Fayer and Liotti [10] found increased emotional interference to physical threat words in individuals with high vs. low levels of anxiety sensitivity using an emotional Stroop task with physical threat, positive and neutral words.

A recent study by Stefan, Zorila and Brie [11] investigated the presence of facilitation, disengagement, or avoidance biases for general-threat and health-related threat stimuli in patients with illness anxiety disorder and in participants with low levels of health anxiety. By means of a spatial cueing task, the authors demonstrated a disengagement bias for health-related threatening stimuli in both patients and low-anxiety individuals. The results suggested that illness anxiety might impact top–down attentional control rather than the early detection of threat, and that, due to the relevance of health in the general population, health-related biases could be found even in healthy individuals.

Hence, at present, the available evidence does not allow one to draw definite conclusions about the impact of health anxiety on the processing of health-related stimuli, also due to differences in the paradigms used and the populations employed. Moreover, health-related valence has never been systematically evaluated before: does it modulate the motor disposition towards the target stimulus, i.e., slowing down or fastening responses to targets? Although several studies demonstrated that emotional stimuli strongly modulate action disposition [12], large differences have been reported in the direction of such modulation (i.e., slowing or speeding participants’ responses) depending on the specific emotional content of the stimulus [13,14].

Finally, to our knowledge, attentional bias to health-related stimuli has never been studied by employing an emotional go/no-go task. This paradigm appears promising in that it explores motor readiness towards a dominant response (go trials) and the ability to overcome such motor readiness and inhibit motor action (no-go trials). 

In particular, in the traditional go/no-go paradigm [15], participants are required to respond as quickly as possible to “go” stimuli, and to withhold responses to “no-go” stimuli. Since “go” are more frequent than “no-go” trials, a prepotent tendency to respond is elicited that must be inhibited for the infrequent “no-go” stimuli. In the emotional go/no-go paradigm, emotional words, faces or pictures are used to test the emotional modulation of response inhibition [16]. The emotional valence of the stimuli can be used as an explicit cue for eliciting the response, for instance, requiring participants to respond to positive stimuli and withhold responding to negative and to neutral ones [17]. Recently, it was demonstrated that, when emotional stimuli are explicitly processed, threatening stimuli can both increase commission errors and speed up participants’ responses [18], thus suggesting that response control in an emotional go/no-go task with threatening stimuli can induce impulsive responding.

The present study employed an emotional go/no-go task to investigate whether response patterns for positive and negative health-related stimuli described in healthy individuals are related to different dispositions to action, which would be reflected, in turn, in greater or lesser difficulty withholding a prepotent motor response. We devised an explicit version of the task to verify the presence of health-related biases during the processing of wellness-related or disease-related information [18] in persons with different levels of health anxiety. To assess health anxiety, we used the Short version of the Health Anxiety Inventory (SHAI [19]), a psychometrically sound tool for measuring health anxiety both in clinical and non-clinical samples [20]. To test to what extent health-related biases were specific to health anxiety, we also assessed participants’ anxiety sensitivity using the Anxiety Sensitivity Index-3 [8,21]. By this means, we could also verify whether safety behaviors in health anxiety, such as avoidance or reassurance seeking [19], could specifically modulate participants’ action disposition towards health-related words. 

Consistent with previous studies [7], we hypothesized that health anxiety and anxiety sensitivity could determine a hypervigilance for health-related information, but with a different perturbation of response control depending on the valence (wellness-related or disease-related) of the stimuli and on the participants’ anxiety-related levels.

## 2. Materials and Methods

### 2.1. Participants

Undergraduate students from the Department of Psychology of the University of Campania Luigi Vanvitelli (Caserta, Italy) were recruited by using the convenience sampling method. To be considered eligible for the study, the participants had to satisfy the following inclusion criteria: (i) normal or corrected to normal eyesight; (ii) no past or current self-reported neurological or psychiatric disease; (iii) and no past or current use of psychoactive medications. All participants were Italian native speakers and were recruited in 2019.

The study was approved by the Institutional Review Board of the Department of Psychology of the University of Campania Luigi Vanvitelli (Caserta, Italy). Before taking part to the study, participants received a complete description of the study procedures in accordance with the Declaration of Helsinki, but they were naïve to the study aims and predictions.

### 2.2. Self-Report Measures

The following measures were administered: the Short version of the Health Anxiety Inventory (SHAI [19]) and the Anxiety Sensitivity Index-3 (ASI-3 [8,21]).

The (SHAI [19]) is a self-report measure of health anxiety, allowing one to evaluate the full range of phenomena related to health anxiety (e.g., disease conviction, perceived vulnerability to illness, fear and worry about illness). The SHAI has 18 items (SHAI-total) that are scored from 0 (no symptoms) to 3 (severe symptoms); the total scale range is 0–54. We also used 4 items evaluating the perception of negative consequences of illness (SHAI-negative consequences subscale; scored 0–3), and two further subscales assessing avoidance behaviors (10 items; SHAI-avoidance) and reassurance seeking (8 items; SHAI-reassurance) both rated on a 9-point scale, anchored every two points. The SHAI-avoidance subscale evaluates the situations that health-anxious individuals typically tend to avoid (e.g., “talking about illness”, “going to a hospital for treatment”). For avoidance, the anchors are: “would not (avoid it)”, “slightly”, “definitely”, “markedly” and “always”. The SHAI-reassurance subscale evaluates how often individuals tend to seek reassurance from different sources (e.g., “friends”, “family doctor”). For the reassurance scale, the anchors are: “never”, “rarely”, “sometimes”, “often”, and “daily”. The alpha coefficients reported in previous studies ranged from good to excellent across samples (α = 0.74–0.96); test–retest reliability was adequate (r = 0.87; for a review [20,22]).

The ASI-3 [8,21] is a 18-item self-report questionnaire including three 6-item subscales: physical (e.g., “it scares me when my heart beats rapidly”), cognitive (e.g., “when my mind goes blank, I worry there is something terribly wrong with me”), and social (e.g., “it scares me when I blush in front of people”) concerns. Participants are asked to indicate the extent to which they agree or disagree with each item on a 5-point Likert scale (0 = very little to 4 = very much). Total scores range from 0 to 72 and are computed by summing the relevant items. Single subscale scores range from 0 to 24. Cronbach’s alphas for total ASI-3 scale and for the three subscales all suggested good reliability (Cronbach’s α: total ASI-3 = 0.90, physical concerns = 0.87, social concerns = 0.81, cognitive concerns = 0.83) in a nonclinical sample [21]. The use of the ASI-3 total score as a measure of the general fear of anxiety is recommended in both clinical and research settings [23].

### 2.3. Go/No-Go Task

Stimuli used in the explicit go/no-go task consisted of 60 words selected from the Italian Affective Norms for Emotional Words list (ANEW-I [24,25]) on the basis of their valence (see Appendix A): 20 negative mental or physical health-related words (e.g., fever, confused), 20 positive mental or physical health-related words (e.g., muscled, relaxed) and 20 neutral words (e.g., edge, field). To confirm that the three category words only differed in terms of valence and arousal but not for length and familiarity, in a preparatory phase of our study, we conducted an ANOVA on the means of length, familiarity, arousal and valence (derived from normative values), demonstrating a significant effect of the valence category only on arousal and valence (Table 1). Bonferroni-corrected paired comparisons demonstrated that three categories differed for valence significantly (all *p* < 0.001) and that both positive and negative words significantly differed from neutral words in terms of arousal (for both *p* < 0.001). No further significant effects emerged (*p* > 0.05). For this reason, all the selected words were included in the task.

Words were presented in blocks, and words within each category were randomly assigned to the blocks. The task consisted of 4 blocks for a total of 80 trials. Each block consisted of 20 words, 10 of which were taken from one valence category and 10 from a different valence category: (i) a first block included positive go stimuli and neutral no-go stimuli; (ii) a second block included positive no-go stimuli and neutral go stimuli; (iii) a third block included negative go words and neutral no-go words; (iv) a fourth block included negative no-go stimuli and neutral go stimuli. Thus, each block included neutral words coupled with either positive (Positive–Neutral Blocks) or negative words (Negative–Neutral Blocks). 

**Figure 1:**
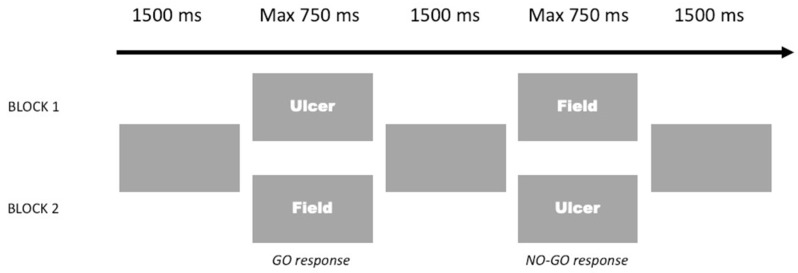
Schematic representation of explicit go/no-go task with examples of Negative–Neutral go and no-go trials.

The trial sequence is displayed in Figure 1. Words were presented in size 30 Courier New font. Each trial began with a grey display presented for 1500 ms, followed by a word. The trial ended immediately after participant’s response, or 750 ms after the appearance of the stimulus if no response was detected. In one block, the participants were asked to respond with a key press on the keyboard to each word belonging to a target valence category (e.g., positive), and to withhold responses to words of the other valence category (e.g., neutral); in the following blocks, opposite associations of response and valence category were alternated. 

Reaction times (RTs), omission errors (no response to go trials) and commission errors (response to no-go trials) were recorded.

### 2.4. Data Analysis

For RTs, only correct responses were considered. The remaining data were fitted with linear mixed-effect modeling [26,27,28,29]. In this kind of modeling, the dependent variable is the sum of both fixed and random effects, with the latter contributing only to data covariance. Mixed modeling relies on single-trial data rather than participants’ averages (or other factors). In this way, random and fixed effects are explicitly controlled. For the present study, linear mixed models were built by means of the “lme4” package [26,27] implemented in R [30]. Statistics for each model were computed by using the “lmertest” package for R [31]. Furthermore, for RTs, the Kenward–Rogers approximation for degrees of freedom was computed. This method works reasonably well with complicated covariance structures and sample sizes ranging from moderate to small [31]. Finally, we ran the “r.squaredGLMM” command (MuMln package) to calculate the conditional and marginal coefficients of determination for generalized mixed-effect models. This command gives two main outputs, namely the marginal coefficient of determination (the variance explained only by fixed factors) and the conditional coefficient of determination (variance explained by both fixed and random factors [32]).

A first model was created in order to investigate the modulation of individual latencies according to the word’s valence (positive, neutral, negative). In such a model, valence was conceived as a fixed effect. 

A second model investigated the extent to which ASI-3 and SHAI predicted the effect of valence. In such a model, the fixed factor valence was put along with the ASI-3 total score and SHAI subscale scores as fixed effects. In both models, participants and items were conceived as random factors. 

As for accuracy, both omission (lack of response to go trials) and commission (response to no-go trials) errors were fitted to the same models as above. However, mixed-effects models were assessed by means of the “glmer” command (“lmer4” package) to deal with binary outcome variables which are modeled as a combination of the predictor variables when data are clustered or there are both fixed and random effects [33]. In each model, the Tukey method for multiple comparisons was applied.

## 3. Results

Thirty-two healthy volunteers were enrolled in this study (25 females; mean age = 24.94 years, SD = 1.88). Data from all participants were included in the analyses. Outliers (4%) were not excluded from the analysis.

### 3.1. Reaction Times

In the first model, with valence as a fixed factor, there was a significant effect of valence (F_1,57_ = 3.34, *p* = 0.04) with faster RTs for positive (mean = 550; SD = 54, 95% Cis = 533–569, *p* = *0*.03) compared to neutral (mean = 572; SD = 38, 95% Cis = 556–590) words. Negative words produced slower RTs (mean = 564; SD = 42, 95% Cis = 547–582) compared to positive and faster RTs compared to neutral, but these differences were not significant (*p* = 0.36; *p* = 0.60, respectively).

In the second model, including the ASI-3 total and the SHAI subscales as fixed factors, the results confirmed the significant main effect of valence (F_2,437_ = 10.51, *p* < 0.001). More importantly, the results showed significant interactions between valence and ASI-3 total (F_3,123_ = 4.51, *p* = 0.004), valence and SHAI-negative consequences (F_3,123_ = 3.83, *p* = 0.01), valence and SHAI-avoidance (F_3,125_ = 6.71, *p* < 0.001), and between valence and SHAI-reassurance (F_3,124_ = 11.43, *p* < 0.001). Results for the two models are summarized in Table 2. 

**Figure 2:**
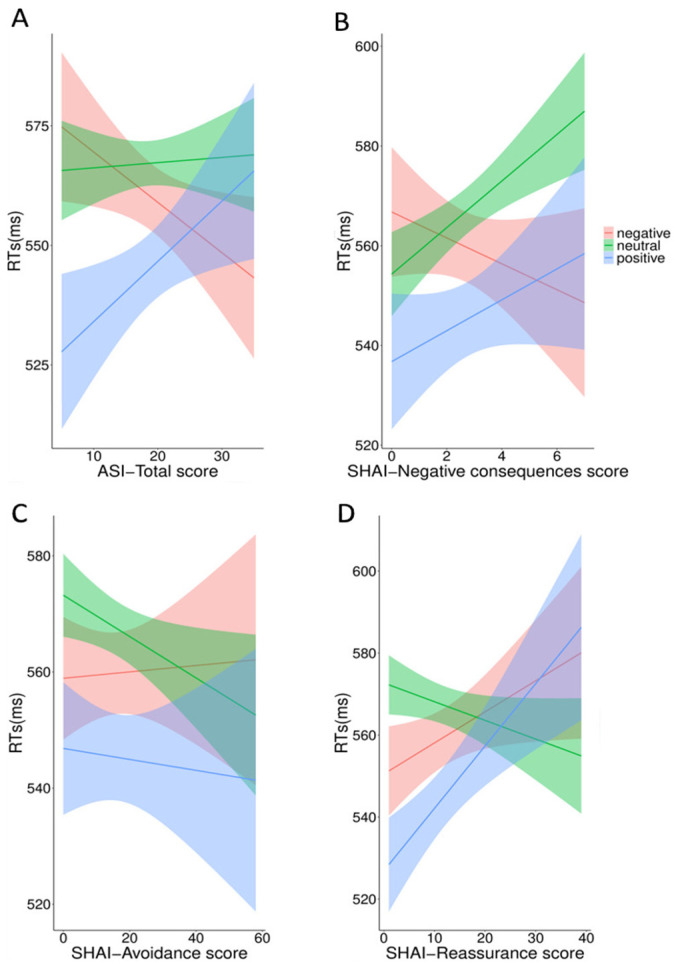
RTs for positive, negative and neutral words as function of the scores at ASI-3 (**A**), SHAI -negative consequences (**B**), SHAI-avoidance (**C**) and SHAI-reassurance (**D**).

Tukey-corrected post hoc analyses revealed divergent trends of positive, neutral and negative words (results are plotted in Figure 2). Although the trends for each valence did not reach the significance level (*p* > 0.05), their slopes were significantly different from each other. As a function of the ASI-3 total score, latencies of positive and neutral words increased (slope positive words = 1.49, 95% Cis = −1.11–4.10; slope neutral words = 1.07, 95% CIs = −1.43–3.58), while those of negative words decreased (slope negative words = −0.95, 95% Cis = −3.54–1.64; positive vs. negative: *p* = 0.003; neutral vs. negative: *p* = 0.004; neutral vs. positive: *p* = 0.79). Latencies of both positive and neutral words showed a similar increase as a function of SHAI-negative consequences (slope positive words = 5.08, 95% CIs = −3.80–13.96; slope neutral words = 5.76, 95% CIs = −2.76–14.28; neutral vs. positive: *p* = 0.94), while latencies of negative words decreased (slope negative words = −1.35, 95% CIs = −10.25–7.55; positive vs. negative: *p* = 0.004; neutral vs. negative: *p* = 0.03). Latencies of neutral and negative words also diverged as a function of the SHAI-avoidance score (*p* < 0.001) showing, respectively, a negative (positive and neutral words: slope positive words = 0.03, 95% CIs = −1.15−1.23; slope neutral words = −0.47, 95% CIs = −1.62–0.67) and a positive trend (negative words, slope = 0.88, 95%CIs = −0.30–2.06). Finally, latencies of positive and negative words increased as a function of the SHAI-reassurance (slope positive words = 1.74, 95% CIs = −0.01–3.49; slope negative words = 1.66, 95% CIs = −0.08–3.40) score and were significantly different from those of the neutral scores (both *p* < 0.001; slope neutral words = −0.39, 95% CIs = −2.06–1.29). No other effects were significant (*p* > 0.05). 

### 3.2. Omission Errors

In the first model with valence as a fixed factor, the results did not show a significant main effect (F_2_ = 0.04, *p* = 0.5). In the second model adding ASI-3 total and SHAI subscales as fixed factors, the results confirmed the results of valence (F_2_ = 0.04, *p* = 0.5). Importantly, the results showed significant interactions between valence and both the ASI-3 total score (F_3_ = 4.17, *p* = 0.003) and SHAI-avoidance (F_3_ = 3.12, *p* = 0.02). The results for the two models are summarized in Table 3.

**Figure 3:**
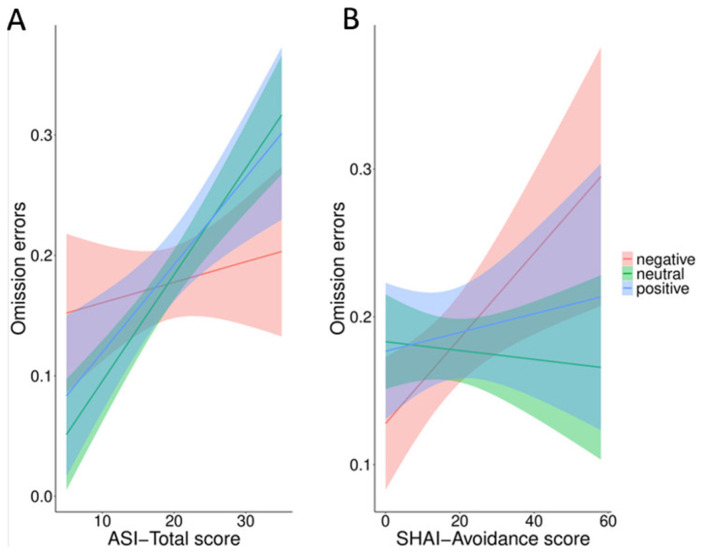
Omission errors for positive, negative and neutral words as function of ASI-3 total score (**A**) and SHAI-avoidance score (**B**).

The results of both interactions are plotted in Figure 3. Tukey-corrected post hoc analysis for the valence by the ASI-3 total score interaction showed that omissions errors significantly increased for positive (slope positive words = 0.01, 95% CIs = −0.05–0.08; *p* = 0.03) and neutral (slope neutral words = 0.07, 95% CIs = 0.01–0.13; *p* = 0.02), but not for negative words (slope negative words = −0.01, 95% CIs = −0.05–0.08; *p* = 0.62) as a function of the ASI-3 total score. Only the difference between neutral and negative words attained significance (*p* = 0.003). 

Tukey-corrected analysis for the valence by SHAI-avoidance interaction showed a higher increase in omissions as a function of the SHAI-avoidance score for negative words as compared to neutral (slope negative words = 0.02, 95% CIs = −0.007–0.05; slope neutral words = −0.01, 95% CIs = −0.03–0.01; *p* = 0.007) and positive words (slope positive words = −0.01, 95%CIs = −0.04–0.02; *p* = 0.02). No other effects were significant (*p* > 0.05).

### 3.3. Commission Errors

In the first model, with valence as a fixed factor, the results showed a significant main effect (F_2_ = 16.50, *p* < 0.001) with less commissions for the neutral (mean = 4%; SD = 5) than for positive (mean = 11%; SD = 12 *p* < 0.001) and negative (mean = 14%; SD = 16, *p* < 0.001) words. Negative words showed more commissions than positive, but this difference did not attain significance (*p* > 0.05).

In the second model, with the ASI-3 total score and SHAI subscale scores added as fixed factors, the results confirmed the significant main effect of valence (F_2_ = 13.71, *p* < 0.001), and highlighted significant interactions of valence with both SHAI-negative consequences (F_3_ = 3.42, *p* = 0.01) and SHAI-reassurance (F_3_ = 3.05, *p* = 0.02). The results are summarized in Table 4. 

**Figure 4:**
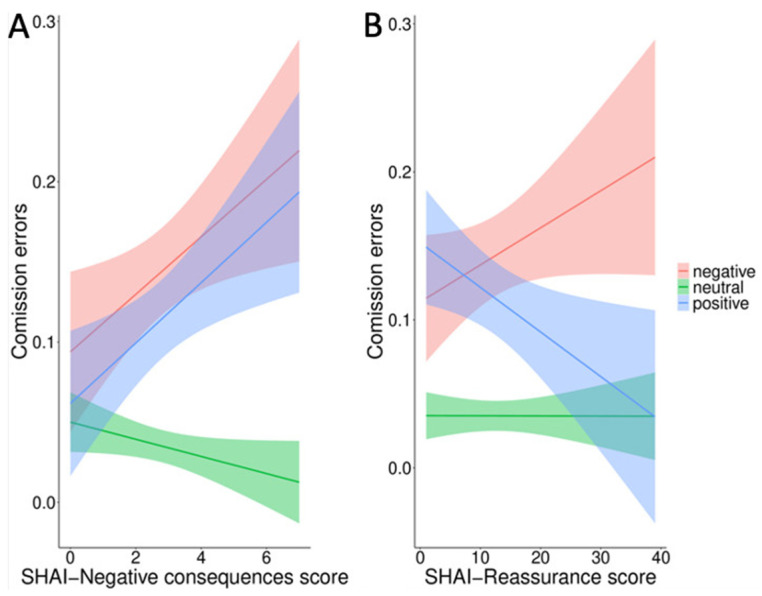
Commission errors for positive, negative and neutral words as function of SHAI-negative consequences (**A**) and SHAI-reassurance scores (**B**).

The results of both interactions are plotted in Figure 4. Tukey-corrected post hoc analysis for the valence by SHAI-negative consequences interaction revealed the same effect for negative and positive words, but not for neutral words. That is, commission errors increased in positive and negative words as a function of the SHAI-negative consequence score (slope positive words = 0.2, 95% CIs = −0.05–0.43; slope negative words = 0.25, 95% CIs = 0.01–0.49). None of these trends were significant (*p* > 0.05), but the slopes of neutral and negative words were significantly different (slope neutral words = −0.1; 95% CIs = −0.39–0.19; *p* = 0.02). 

Tukey-corrected analysis for the valence by SHAI-reassurance interaction showed two divergent trends for positive and negative words (slope positive words = –0.04, 95% CIs = −0.09–0.01; slope negative words = 0.03, 95% CIs = −0.01–0.08). None of these trends were significant (*p* > 0.05), but commission errors were more likely to occur for negative words as a function of the SHAI-reassurance score. Conversely, the same errors decreased for positive words as a function of SHAI-reassurance. The difference was significant (*p* = 0.01). Commission errors for neutral words remained steady (slope neutral words = −0.01; 95% CIs = −0.09–0.01), but they were not significantly different from the trend of negative and positive words (*p* > 0.05). No other effects were significant (*p* > 0.05). 

## 4. Discussion

In the present study, we used an emotional go/no-go task to investigate action dispositions in response to wellness-related or disease-related stimuli and the relationship between response patterns and levels of health anxiety and anxiety sensitivity in healthy individuals.

The main results showed that (1) participants were significantly faster to respond to positive than neutral stimuli, and both negative and positive stimuli determined more commission errors than neutral stimuli; (2) both health anxiety and anxiety sensitivity affected the ability to respond to positive and negative stimuli, but with different patterns; (3) health-related safety-seeking and avoidance behaviors affected action disposition differently in response to positive and negative stimuli. These findings supported our starting hypothesis that health anxiety and anxiety sensitivity could determine a hypervigilance for health-related information with a different pattern of response control depending on the valence of the stimuli.

Faster responses to positive than neutral stimuli were previously reported in the literature [18,34], together with more commission errors for both positive and negative stimuli compared to neutral stimuli [16,35,36]. Our results completely fit this pattern, as faster responses to positive stimuli were coupled with a higher rate of commission errors for both positive and negative stimuli compared to neutral stimuli. According to a motivational approach–withdrawal hypothesis [37], positive emotions, such as happiness and surprise, are related to approaching behaviors, whereas negative emotions, such as sadness and fear, are associated with withdrawal behaviors. From this perspective, our findings of fast RTs and high commission errors for wellness-related stimuli could be consistent with a natural tendency to approach positive stimuli. However, we also found higher commission errors for negative, disease-related stimuli, in line with the hypothesis of a reduced ability to inhibit responses to threatening stimuli due to a reduced efficiency in cognitive control [38,39].

More relevant here, we found that an individual’s sensitivity to health and anxiety issues significantly influenced the response control. Indeed, increased anxiety sensitivity was related to faster RTs and a lower omission rate for negative than for positive and neutral words. A previous study revealed that anxiety-related dysfunctional beliefs measured by ASI-3 predicted health concerns in patients with panic disorders and major depression disorder [40]. Another study [41] also demonstrated that the dimensions of anxiety sensitivity (e.g., physical concerns) predicted some aspects of health anxiety (e.g., body vigilance) more strongly than others (e.g., illness severity) in healthy individuals. Clark [42] suggested that the tendency to interpret arousal-related sensations catastrophically could lead to maladaptive responses, as in panic attacks. In our study, anxiety-related dysfunctional beliefs were tied to approaching action tendencies (faster RTs and reduced omissions) towards negative stimuli. On this basis, we could suggest that the fear of anxiety symptoms and associated catastrophic consequences in individuals with a high level of anxiety sensitivity implies the prioritized processing of health-related threatening information. However, in the present study, no commission errors were related to the ASI-3 total score, a finding that seems at odds with this idea. As commissions were significantly influenced by levels of health anxiety, interference with action control seems a prerogative of health anxiety rather than of anxiety sensitivity. Indeed, a larger number of commission errors to both positive and negative words was related with higher SHAI-negative consequences subscale scores and a larger number of commission errors to negative words only with higher SHAI-reassurance subscale scores.

The SHAI-negative consequences subscale requires participants to think about what it might be like if they had a serious illness (such as heart disease, or cancer). People have to estimate what might happen considering what they know about themselves and about the illness in general. Thus, this subscale evaluates the catastrophic expectation regarding the negative consequences, i.e., the burden and outcome, of having a serious illness. Salkovskis et al. [19] compared patients with health anxiety disorders to patients with other anxiety disturbances and found that the negative consequences of the illness subscale were highly specific for patients with health anxiety as compared with other anxious groups. Since people experiencing persistent anxiety about health have a systematic tendency to misinterpret ambiguous medical information in a negative way [19], they are captured by both negative and positive words, as shown here by both faster RTs and commission errors.

Persons with high scores on SHAI-reassurance show different action tendencies towards health-related stimuli. The SHAI-reassurance subscale evaluated how often individuals tend to seek reassurance from different sources (e.g., friends, or a family doctor). Repeated medical consultations and tests, self-checking, compulsive requests for reassurance and repeated searches for reassuring information are evidence of dysfunctional safety behaviors that can reinforce health anxiety. Here, slower RTs for both positive and negative words with respect to neutral words were found in individuals with a stronger tendency to seek reassurance, whereas commission errors were more likely to occur specifically for negative words. These results could suggest that positive health-related stimuli did not interfere with action control, whereas task-relevant negative health-related stimuli induced impulsive responses in relation with higher SHAI-reassurance scores. Actually, an increase in commission errors without a parallel speeding of RTs would not be consistent with the hypothesis that threat-induced commissions were related to a shift towards speed at the expense of accuracy [43]. On this basis, we could suggest that in individuals who tend to seek reassurance, increased commission errors could represent an effect of increased vigilance toward self-relevant stimuli (an attentional bias) rather than of increased impulsivity towards threatening stimuli. 

Finally, we found that higher levels of health anxiety-related avoidance behaviors, measured with the SHAI-avoidance subscale, were related to slower RTs and increased omissions for negative than for positive and neutral words. These results fit the idea that threatening health-related stimuli can induce withdrawal responses in individuals who tend to avoid negative health-related information. The SHAI-avoidance subscale evaluates the situations that health anxious individuals typically tend to avoid (e.g., talking about illness, or going to a hospital for treatment). Generally, avoiding unpleasant or negative stimuli are considered a core element of adaptive behavior [44]. Nevertheless, the maladaptive avoidance of aversive or generally negative information is considered to be implicated in various mental disorders (for a review, see [45]). Avoidance, as well as other safety behaviors, is shown by individuals with health anxiety disorder and other anxiety disorders [46]: individuals attempt to cope with threats to their health by avoiding hospitals, specialist clinics and sick people. Such avoidance behaviors prevent one from experiencing situations and gaining information that could disconfirm maladaptive beliefs about one’s health conditions. Thus, avoidance behaviors increase the estimations of the probability of suffering from some disorder and increase health anxiety, and other anxiety disturbances such as panic disorder [19].

One main limitation of the present study is that our sample was selected without computing the sample size and included a small percentage of male individuals. Since the unbalanced sample could reduce the generalizability of our results, our findings need to be verified in further studies on larger and balanced samples. Moreover, we employed a convenience sample of healthy participants; it would have been important to assess the prioritization of health-related information as a function of different safety behaviors in individuals with illness anxiety disorder. Further studies including a clinical population are necessary in order to develop innovative assessment and treatment tools. A further consideration should be made on the specific task we used here. Analyzing the pattern of results obtained on an emotional go/no-go task, Schulz et al. [16] suggested that commission errors on no-go trials could provide a measure of behavioral inhibition, whereas differences in reaction times across go trials with cues of different emotional values could offer an index of emotional preference (i.e., bias). Nevertheless, the explicit go/no-go task used in the present study may have triggered a process of response selection [47,48,49] since the participants had to choose whether to respond or not based on the valence of the stimuli. Indeed, in our explicit task, we required participants to choose if the word was “negative” or “positive” and then to respond or not. This could have determined longer RTs necessary for assessing the valence of the word and for the response execution decision. Further studies could use an implicit version of this task or a different task for assessing motor control.

The present findings pave the way for employing new assessment tools to complement classical self-report assessment measures and allow one to hypothesize treatment interventions in which action readiness to health-related stimuli might be progressively inhibited, analogously to procedures employed for modification of attentional biases. Studies on attentional bias modification training (ABMT [50]) in clinical populations employed a dot-probe paradigm [51] to train individuals to divert their attention away from threat. Experiments on patients with anxiety disorders [52] demonstrated that extensive repetitions of such trials induce an implicitly learned bias away from threatening stimuli [53]. ABMT was proven to be useful in reducing anxiety and depressive symptoms in individuals not responsive to cognitive behavioral therapy [54]. The present findings could help to implement similar training procedures for reducing potentially dysfunctional responses to relevant health-related stimuli in persons with health anxiety.

Finally, recent evidence [55] demonstrated that by applying an inhibitory transcranial direct current stimulation (tDCS) over the right temporoparietal junction, it is possible to modulate attention, wiping out the difference between responses to threatening and non-threatening stimuli. An integrated approach of behavioral training and tDCS protocols could be used to reduce biases and anxiety symptoms in health-related anxiety disorders.

## 5. Conclusions

Taken together, our study suggested that health anxiety and anxiety sensitivity could lead to the different prioritization of health-related stimuli, and that health-related safety-seeking and avoidance behaviors can differently affect action tendencies towards wellness-related and disease-related stimuli. Since health anxiety and health anxiety disorder do form a continuum, capturing different action biases to health-related stimuli could improve the detection of processing biases in persons who might develop a clinical condition.

## Figures and Tables

**Table 1:** Stimulus statistics with mean, range and standard deviation (SD) for negative, neutral and positive words employed; valence, arousal, length and familiarity from ANEW-I database (Bradley and Lang, 1999).

	Negative	Positive	Neutral	Fixed Factors Statistics	
Characteristic	Mean [Range] (SD)	Mean [Range] (SD)	Mean [Range] (SD)	F	*p*
Valence	2.25 [2–3] (0.44)	7.75 [7–9] (0.5)	5.25 [5–6] (0.44)	652.45	<0.001
Arousal	6.35 [4–8] (0.93)	6.3 [4–8] (1.03)	4.9 [4–6] (0.55)	18.15	<0.001
Length	7.3 [5–8] (1.84)	7.3 [5–9] (1.84)	7.3 [6–8] (1.84)	0	1
Familiarity	6.45 [4–11] (1)	6.85 [4–11] (0.81)	6.85 [4–11] (0.74)	1.45	0.24

**Table 2:** M1 investigates the main effect of valence (positive vs. neutral vs. negative) on RTs. M2 tests the interaction between valence and ASI and SHAI’s subscales scores.

Model	Fixed Factors	Fixed Factors Statistics		Model’s Statistics			
		F	*p*	AIC	BIC	r^2^_m_	r^2^_c_
M1	Valence	3.35	0.04	24,000	24,034	0.01	0.27
M2	Valence	10.51	<0.001	23,952	24,071	0.05	0.33
	Valence × ASI-Total	4.51	0.004				
	Valence × SHAI-Total	1.49	0.22				
	Valence ×SHAI -Avoidance	6.71	<0.001				
	Valence × SHAI-Reassurance	11.43	<0.001				
	Valence × SHAI-Negative Consequences	3.83	0.01				

**Table 3:** M1 investigates the main effect of valence (positive vs. neutral vs. negative) on omission errors. M2 tests the interaction between valence and ASI and SHAI’s subscales scores.

Model	Fixed Factors	Fixed Factors Statistics		Model’s Statistics			
		F	*p*	AIC	BIC	r^2^_m_	r^2^_c_
M1	Valence	0.4	0.5	2075	2104	0.0003	0.35
M2	Valence	0.4	0.5	2065	2182	0.05	0.37
	Valence × ASI-Total	4.17	0.003				
	Valence × SHAI-Total	2.08	0.07				
	Valence × SHAI-Avoidance	3.12	0.02				
	Valence × SHAI-Reassurance	1.07	0.22				
	Valence × SHAI-Negative Consequences	2.57	0.29				

**Table 4:** M1 investigates the main effect of valence (positive vs. neutral vs. negative) on commission errors. M2 tests the interaction between valence and ASI and SHAI’s subscales scores.

Model	Fixed Factors	Fixed Factors Statistics		Model’s Statistics			
		F	*p*	AIC	BIC	r^2^_m_	r^2^_c_
M1	Valence	16.50	<0.001	1264	1293	0.11	0.38
M2	Valence	13.71	<0.001	1262	1379	0.20	0.43
	Valence × ASI-Total	0.94	0.88				
	Valence × SHAI-Total	1.58	0.33				
	Valence × SHAI-Avoidance	0.68	0.56				
	Valence × SHAI-Reassurance	3.05	0.02				
	Valence × SHAI-Negative Consequences	3.42	0.01				

## Data Availability

The conditions of our ethics approval do not permit public archiving of anonymized study data. Readers seeking access to the data should contact the lead author, Laura Sagliano at the Department of Psychology, University of Campania Luigi Vanvitelli. Access can be granted only to named individuals in accordance with ethical procedures governing the reuse of sensitive data.

## Appendix A

**Table A1:** Stimuli used in the emotional go/no-go task.

Words in Italian	Words in English	Valence	Arousal	Length	Familiarity	Valence Category
Muco	Mucus	3	5	4	8	Negative
Cieco	Blind	2	6	5	5	Negative
Morte	Death	2	7	5	7	Negative
Dolore	Ache	2	7	6	7	Negative
Ulcera	Ulcer	2	6	6	5	Negative
Febbre	Fever	3	6	6	7	Negative
Panico	Panic	2	7	6	6	Negative
Stress	Stress	2	8	6	8	Negative
Confuso	Confused	3	6	7	7	Negative
Malanno	Illness	2	6	7	7	Negative
Ansioso	Anxious	2	7	7	7	Negative
Ospedale	Hospital	3	7	8	7	Negative
Affogare	To Drown	2	8	8	5	Negative
Paralisi	Paralysis	2	7	8	5	Negative
Ambulanza	Ambulance	3	6	9	8	Negative
Emicrania	Migraine	2	4	9	6	Negative
Infezione	Infection	2	6	9	6	Negative
Malessere	Malaise	2	6	9	6	Negative
Angosciato	Distressed	2	6	10	6	Negative
Depressione	Depression	2	6	11	6	Negative
Sexy	Sexy	8	8	4	7	Positive
Gioia	Joy	9	7	5	7	Positive
Forte	Strong	7	7	5	7	Positive
Salute	Health	8	5	6	7	Positive
Estasi	Ecstasy	7	7	6	6	Positive
Sereno	Serene	8	5	6	7	Positive
Gloria	Glory	7	6	6	6	Positive
Risata	Laugh	8	6	6	9	Positive
Allegro	Cheerful	8	6	7	7	Positive
Agilità	Agility	8	7	7	6	Positive
Orgasmo	Orgasm	8	7	7	7	Positive
Bellezza	Beauty	8	7	8	7	Positive
Paradiso	Paradise	8	5	8	5	Positive
Vigoroso	Vigorous	7	6	8	6	Positive
Ottimismo	Optimism	8	6	9	7	Positive
Godimento	Enjoyment	8	6	9	7	Positive
Muscoloso	Muscled	7	6	9	7	Positive
Rilassato	Relaxed	8	4	9	7	Positive
Entusiasmo	Enthusiasm	8	8	10	8	Positive
Eccitazione	Excitement	7	7	11	7	Positive
Comò	Commode	5	4	4	8	Neutral
Bordo	Edge	5	5	5	6	Neutral
Campo	Field	6	5	5	7	Neutral
Angolo	Angle	5	5	6	7	Neutral
Fanale	Light	5	5	6	6	Neutral
Roccia	Rock	5	5	6	7	Neutral
Vicolo	Alley	5	5	6	6	Neutral
Sedile	Seat	6	4	6	8	Neutral
Tappeto	Carpet	6	5	7	7	Neutral
Sughero	Cork	5	4	7	7	Neutral
Fischio	Whistle	5	6	7	7	Neutral
Finestra	Window	6	4	8	8	Neutral
Gelatina	Jelly	5	5	8	6	Neutral
Opuscolo	Brochure	6	5	8	6	Neutral
Ascensore	Lift	5	5	9	8	Neutral
Bollitore	Kettle	5	5	9	6	Neutral
Manichino	Dummy	5	5	9	7	Neutral
Serbatoio	Tank	5	5	9	7	Neutral
Pizzicotto	Tweaks	5	6	10	7	Neutral
Sciocchezza	Foolishness	5	5	11	6	Neutral
